# Future Treatment Options in Systemic Sclerosis—Potential Targets and Ongoing Clinical Trials

**DOI:** 10.3390/jcm11051310

**Published:** 2022-02-27

**Authors:** Anna Bohdziewicz, Katarzyna Karina Pawlik, Magdalena Maciejewska, Mariusz Sikora, Rosanna Alda-Malicka, Joanna Czuwara, Lidia Rudnicka

**Affiliations:** 1 Department of Dermatology, Medical University of Warsaw, Koszykowa 82A, 02-008 Warsaw, Poland; bohdziewicz99@gmail.com (A.B.); katarzynapawlik97@gmail.com (K.K.P.); rosie_alda@msn.com (R.A.-M.); jczuwara@yahoo.com (J.C.); lidiarudnicka@gmail.com (L.R.); 2 National Institute of Geriatrics, Rheumatology and Rehabilitation, Spartańska 1, 02-637 Warsaw, Poland; drmariuszsikora@gmail.com

**Keywords:** systemic sclerosis, riociguat, belimumab, SAR100842, tofacitinib, nilotinib, imatinib, nintedanib, brentuximab vedotin, rituximab

## Abstract

Systemic sclerosis is an autoimmune connective tissue disease characterized by vasculopathy and fibrosis of the skin and internal organs. The pathogenesis of systemic sclerosis is very complex. Mediators produced by immune cells are involved in the inflammatory processes occurring in the tissues. The currently available therapeutic options are often insufficient to halt disease progress. This article presents an overview of potential therapeutic targets and the pipeline of possible future therapeutic options. It is based on research of clinical trials involving novel, unestablished methods of treatment. Increasing knowledge of the processes and mediators involved in systemic scleroderma has led to the initiation of drug trials with therapeutic targets of CD28-CD80/86, CD19, CCL24, CD20, CD30, tumor necrosis factor (TNF), transforming growth factor β (TGF-β), B-cell activating factor (BAFF), lysophosphatidic acid receptor 1 (LPA1 receptor), soluble guanylate cyclase (sGC), Janus kinases (JAK), interleukin 6 (IL-6), endothelin receptor, and autotaxin. Data from clinical trials on these drugs indicate a significant potential for several new therapeutic options for systemic sclerosis in the upcoming future.

## 1. Introduction

The pathogenesis of systemic sclerosis (SSc) is complex and involves a variety of genetic and unidentified sophisticated environmental factors. Numerous pieces of evidence indicate an interplay between the immune system, especially T and B cells, and fibroblasts, leading to fibrosis. The discovery of processes and mediators involved in the pathogenesis of SSc will help to provide new therapeutic targets, and may contribute to the development of new potentially effective drugs. Clinical experience has proven that disease modification remains intangible, and treatment is focused on systemic sclerosis complications, such as interstitial lung disease (ILD), skin fibrosis, pulmonary artery hypertension, and peripheral vasculopathy [1]. Early treatment of SSc is most effective; thus, early diagnosis is crucial. However, this may be very difficult as the majority of typical signs are absent in the early stages. Another issue is the absence of reliable biomarkers that predict disease evaluation [2]. This review aims to give an overview of current prospects for possible therapeutic targets, and the ongoing research to find an efficient drug in SSc treatment. Findings from recent studies found via electronic resources are summarized, focusing especially on the efficacy and safety of biologic agents and other novel medications.

## 2. Materials and Methods

A review of the literature published from January 2006 to December 2021 was performed using Pubmed database. A search was conducted utilizing MeSH terms: “systemic sclerosis” or “monoclonal antibodies” or “biotherapies in systemic sclerosis” or “scleroderma” or “systemic sclerosis management” and “systemic sclerosis treatment”. Ongoing clinical trials were searched through the ClinicalTrials database. Among the results, trials related to established methods of treatment were excluded.

## 3. Results

Possible therapeutic targets and related medications are presented in Table 1, which also provides a summary of the latest research included in this review (Table 1).

### 3.1. CD28-CD80/86 (Cluster of Differentiation 28—Cluster of Differentiation 80/86)

T cells play a key role in the pathogenesis of SSc, and the activation and proliferation of naïve T cells requires co-stimulatory signals. One of the most relevant co-stimulatory tracts is in the CD28-CD80/CD86 pathway [3]. Abatacept (ABA) is a recombinant CTLA4-Ig fusion protein, binding to CD80/CD86 on antigen presenting cells (APCs), and blocking CD28 binding to CD80/CD86, which results in the suppression of T cell activation. The results of a retrospective multicenter observational study with 27 SSc patients enrolled suggest that ABA has a good safety profile. Most adverse effects occurred in the first 3 months after ABA application. The main indication to use ABA was joint involvement. After 12 months of use, the number of swollen and tender joints decreased compared to the baseline (*p* < 0.03 and *p* < 0.02, respectively). A decrease in the modified Rodnan skin score (mRSS) was also observed (*p* < 0.05). These findings suggest that ABA may be useful in patients with musculoskeletal involvement [3]. However, according to the results of a phase II investigator-initiated, multicenter, double-blind, randomized, placebo-controlled trial, ABA is well tolerated; however, the change in mRSS seems to not be statistically significant. There is a need to conduct a phase III trial before assessing the definitive efficacy and safety of ABA in diffuse cutaneous SSc [4].

### 3.2. CD19 (Cluster of Differentiation 19)

CD19 is expressed on the surface of pro-B cells and on all B cell lineages. Indeed, CD19 is observed very early in B cell development. It is worth highlighting that a significant proportion of plasmablasts and plasma cells also have CD19 on the surface [5]. MEDI-551 is a humanized monoclonal antibody against CD19, which mediates antibody-dependent, cell-mediated cytotoxicity of B cells. The main aim of a phase I multicenter, randomized, double-blind, placebo-controlled, single escalating dose study was to evaluate the safety and tolerability, pharmacokinetics, and pharmacodynamics of MEDI-551 in subjects with SSc. The results of this 28-patient study indicate a depletion of circulating B cells and plasma cells, as well as a possible clinical effect on affected skin, measured by mRSS. In addition, MEDI-551 seems to have a good safety profile. The assessment includes the incidence of adverse events and changes in clinical and laboratory results. Indeed, further studies are needed to prove that MEDI-551 may be a potential disease-modifying drug for SSc [6].

### 3.3. CCL24 (Chemokine C-C Motif Ligand 24, Eotaxin 2)

CCL24 is a known chemoattractant for eosinophils through its receptor CCR3. Some data indicate that IL-4 and IL-10 stimulate macrophages to produce a large amount of CCL24 and consequently increase eosinophil migration. CCL24 is also considered to take part in proinflammatory reactions and to promote collagen production by human lung fibroblasts. Elevated levels of CCL24 and increased expression of CCR3 have been observed in SSc patients when compared with healthy patients [7]. CM-101 is a humanized monoclonal antibody targeting the human chemokine CCL24. The results of the study, in which the main aim was to assess the ability of CCL24-blocking antibody CM-101 to interfere with profibrotic activities induced by CCL24, in both in vitro and in vivo models of skin and lung fibrosis, are promising. Their findings suggest that CM-101 significantly inhibits endothelial cell activation induced by CCL24. Moreover, skin and lung fibrosis and inflammation were suppressed by CM-101 in bleomycin (BLM)-induced models of fibrosis [7].

### 3.4. CD20 (Cluster of Differentiate 20)

Rituximab (RTX) is a monoclonal antibody against CD20 that eliminates B cells which, in turn, are considered key players in the fibrotic process. In recent years, the depletion of B cells has become a potential therapeutic strategy for SSc, especially for lung and skin fibrosis [8]. Results of a study conducted in Japan suggest that, for patients with SSc-associated interstitial lung disease (SSC-ILD), RTX treatment is more effective than cyclophosphamide (CYC) therapy, which is one of the current therapeutic options for SSc-ILD patients [9]. Moreover, RTX was shown to have a beneficial effect on lung function as well as on skin fibrosis according to the results of a multicenter study, in which 51 SSc-ILD patients participated and were divided into two groups. One group was treated with RTX (33 patients), and the other group with conventional therapy (18 patients): azathioprine (2 patients), methotrexate (6 patients), and mycophenolate mofetil (10 patients, dosage 2 g/d), as well as small doses of corticosteroids (17/18 patients). Indeed, the same study revealed that RTX was generally well tolerated and that adverse events were comparable between the groups [8]. Interestingly, a 52-week, single-center, randomized, double-blind, placebo-controlled study is now ongoing. Specific objectives of this study are: to determine whether rituximab/belimumab/mycophenolate mofetil (MMF) is safe and tolerable, and to assess whether these medications are more effective than placebo/placebo/MMF measured by change in composite response index in diffuse cutaneous systemic sclerosis (CRISS). The estimated primary completion year is 2022 [10]. It is also worth mentioning that a multicenter, double-blind, placebo-controlled, parallel-group comparison, investigator-initiated clinical trial was performed to evaluate the efficacy and safety of rituximab for SSc patients. In this trial, 56 participants were randomly assigned to one of two groups—the placebo group or the rituximab group. The absolute change in mRSS 24 weeks after the start of the intervention, which was the primary endpoint, was lower in the rituximab group than in the placebo group [11]. In addition, the %FVC of 48 patients with ILD at 24 weeks was improved in the rituximab group more so than in the placebo group (the change in %FVC at 24 weeks was the secondary endpoint). The most common adverse event was upper respiratory infections [12].

### 3.5. CD30 (TNFRSF8—Tumor Necrosis Factor Receptor Superfamily Member 8)

Brentuximab Vedotin targets the protein CD30 expressed on activated immune cells, particularly lymphocytes. The great majority of patients with dcSSc have elevated levels of soluble CD30 in their sera, which suggests that patients with progressive systemic sclerosis have predominant activation of Th2-like cells [13]. CD30 targeting has been shown to produce growth inhibition and apoptosis in immune cells. It is important to mention that two clinical trials are now ongoing to evaluate the therapeutic efficacy of brentuximab vedotin in patients with active diffuse cutaneous SSc (dcSSC). The estimated primary completion years were supposed to be 2021 and 2020; however, no results have been posted yet [14,15].

### 3.6. TNF (Tumor Necrosis Factor)

Tumor necrosis factor (TNF) belongs to a group of proinflammatory cytokines possessing pleiotropic effects on a variety of cells. It is also involved in the pathogenesis of inflammatory diseases. It has been reported that serum levels of TNF were increased in SSc patients compared to healthy controls [16]. Infliximab is a chimeric human/murine IgG1 monoclonal antibody targeting TNF. According to the specific literature assessing the safety and efficacy of TNF inhibitors, infliximab has beneficial effects on the inflammatory arthritis and disability associated with SSc [17]. The findings of the study, which enrolled 16 dcSSc patients, demonstrated that there was no relevant change in mRSS at week 26. However, infliximab was associated with clinical stabilization and a fall in two markers of collagen synthesis. There were no deaths during the study and no unexpected adverse reactions [18]. Adalimumab is a human monoclonal antibody targeting TNF. It was reported that adalimumab leads to reduction in a chemerin levels. Chemerin is considered a biomarker of renal involvement in SSc patients. A reduction in other pro-inflammatory cytokine levels was also seen [19]. It is important to mention that adalimumab and infliximab may induce the secretion of human anti-infliximab and anti-adalimumab antibodies (neutralizing or non-neutralizing). This immune reaction is the main factor limiting the treatment with TNF inhibitors because neutralizing antibodies, among others, inhibit the possibility of achieving a clinical remission or a minimal disease activity state. It was reported that the supplementary administration of disease-modifying antirheumatic drugs (DMARDs), such as methotrexate or leflunomide, prevents the development of neutralizing Abs against these TNF inhibitors [20]. Etanercept is a fusion protein that acts as a ‘decoy receptor’ for TNF [21]. It was indicated that etanercept improves the inflammatory arthritis and disability associated with SSc [16]. Iguratimod (IGU), a novel small-molecule anti-rheumatic drug that is approved in Japan and China, is a methane sulfonanilide that is chemically composed of (N-[7-[(methanesulfonyle)amino]-4-oxo-6-phenoxy-4H-1-benzopyran-3-yl]-formamide). Pharmacological studies have shown that IGU can inhibit the production of various inflammatory cytokines, including interleukin IL-1, IL-6, IL-8, and tumor necrosis factor (TNF). Recent data reveal its independent anti-fibrotic effect [22,23]. A study will be started soon to evaluate the safety, tolerability, and efficacy of IGU in adult subjects with diffuse cutaneous systemic sclerosis [24].

### 3.7. TGF-β (Transforming Growth Factor β)

Transforming growth factor β (TGF-β) has potent profibrotic activity, and promotes collagen synthesis, secretion, processing, and cross-linking [25]. All isotypes of TGF-β (TGF-β1, TGF-β2, and TGF-β3) have an interaction with the same receptor complex and demonstrate a specific effect on cell proliferation and differentiation [26]. Fresolimumab is a human monoclonal antibody that targets all 3 TGF-β isoforms. An open label trial was conducted of which the main aim was to elucidate the specific effect of TGF-β pathway blockage by fresolimumab. Of the 15 patients enrolled, 7 patients received 1 mg/kg doses of fresolimumab and 8 patients received one 5 mg/kg dose of fresolimumab. Skin biopsies were performed before and after treatment to assess the expression of TGF-β-regulated genes, while the clinical skin response was evaluated using mRSS. The sudden inhibition of TGF-β-regulated gene expression in response to the targeted treatment implicates TGF-β in the pathogenesis of fibrosis in SSc. A parallel improvement in mRSS was also indicated [26].

### 3.8. BAFF (B Cell Activating Factor)

B cell activating factor (BAFF) plays a role in the proliferation, differentiation, and activation of B cells. The stimulation of collagen synthesis by B cells contributes to fibrosis in SSc. An elevation in the serum levels of BAFF in SSc patients was observed which is, moreover, associated with organ involvement and skin fibrosis [27]. Belimumab is a monoclonal antibody that inhibits BAFF. It is already accepted to treat lupus erythematosus. A 52-week, investigator-initiated, single-center, double-blind, placebo-controlled, pilot study took place to assess the safety and efficiency of belimumab in patients with early diffuse cutaneous systemic sclerosis (dcSSc) on a background of MMF therapy. Patients treated with belimumab showed a greater improvement in mRSS; however, this was not statistically significant. A significant change in gene expression only occurred in the belimumab group, in whom improvement was observed. There was a decreased signaling of B cells, fibrotic genes, and pathways (including collagens, free extracellular matrix proteins, and TGFB/TGFBR). The adverse events were comparable in the study and the control group treated with placebo. To investigate the direct impact of belimumab, studies on patients treated only with belimumab are required. However, there is reason for further research [28].

### 3.9. LPA1 Receptor (Lysophosphatidic Acid Receptor1)

LPA stimulates cell division as well as differentiation of lung fibroblasts to myofibroblasts. A similar process takes place in the pathogenesis of SSc. It was investigated that, in skin biopsies and blood samples from SSc, patients’ levels of LPA 1 are higher than in healthy individuals [29]. SAR100842 is a selective oral antagonist of the LPA1 receptor. An 8-week, phase 2a study with an open-label extension for 16 weeks was conducted to investigate the effects and treatment tolerance of SAR100842 in patients with dcSSc. SAR100842 showed good tolerability. A greater reduction in mRSS and LPA-related genes was observed in the skin of the SAR100842 group compared to the placebo, although it did not prove significant. A larger control trial is necessary to evaluate the results [30].

### 3.10. sGC (Soluble Guanylate Cyclase)

Stimulators of soluble guanylate cyclase (sGC) have been shown to block transforming growth factor-β signaling and to inhibit fibrosis. They could be relevant for the treatment of fibrosis and vascular disease in systemic sclerosis [31]. Riociguat is a soluble guanylate cyclase (sGC) modulator that is investigated as a potential medication in systemic sclerosis. It is already approved for pulmonary arterial hypertension, which often accompanies scleroderma. Riociguat has both vasoactive and antifibrotic effects. RISE-SSc, a randomized, double-blind, placebo-controlled phase 2 study investigating riociguat in patients with diffuse cutaneous systemic sclerosis, was conducted. Patients did not experience any unexpected adverse events. Gastrointestinal events, dizziness, and peripheral oedema were more frequent in the riociguat group compared to the placebo group. However, overall, treatment was well tolerated. During the trial, forced vital capacity declined by 2.7% with riociguat and 7.6% with placebo. Nevertheless, the decrease in mRSS was not statistically significant [32]. Another study was made to determine the effect of riociguat on the net digital ulcer (DU) burden in systemic sclerosis (SSc) [33]. There was no significant treatment difference observed in the change from baseline to 16 weeks in net ulcer burden (NUB). In the open-label extension in patients treated with riociguat, DUs were healed. This suggests that more time is needed to heal the ulcers; however, this needs to be confirmed in a new trial. Overall, small potential efficacy signals have been shown in secondary analyses that should be tested in further trials [32,33].

### 3.11. JAK (Janus Kinases)

The amount of evidence on the JAK/signal transducer and activator of transcription (STAT) pathways, which participate in pathological processes in autoimmune diseases, is increasing. Therefore, interest in inhibitors of this pathway has grown. Multi-targeted cytokine signal inhibition makes it possible for these to be effective in the treatment of SSc [34]. In the phase I/II trial, 15 participants were randomized to assess the safety of tofacitinib in patients with dcSSc. Tofacitinib was well tolerated. Manageable adverse effects were observed. There were trends toward improvements in clinical outcome measures, such as mRSS. According to this study, further evaluation of tofacitinib in dcSSc is warranted [35]. Peficitinib, a novel JAK inhibitor, was the subject of an in vitro study which was made to characterize the pharmacological properties of peficitinib, and to investigate the involvement of JAK/STAT pathways in SSc. Results suggest that JAK inhibitors, such as peficitinib, may represent a new therapeutic option for SSc [33]. A novel study is about to start in 2022 assessing Itacitinib is a JAK inhibitor that specifically targets JAK1 and decreases STAT3 phosphorylation [36].

Tyrosine kinases are utilized by the signaling cascades of the profibrotic cytokines, such as transforming growth factor-β (TGF) and platelet-derived growth factor (PDGF). According to preclinical studies, their inhibitors could be potentially effective in treating fibrosing disorders [37]. Imatinib mesylate is an inhibitor of tyrosine kinase, known by most doctors for its use in the therapy of chronic myelogenous leukemia and gastrointestinal stromal tumors. It was reported that mRSS and FVC significantly improved in dcSSc patients who were treated with imatinib. The increase in FVC was larger in patients without ILD. The analysis confirmed a significant decrease in skin thickness and improvement in its morphology. Adverse effects were quite common; however, overall, treatment was well tolerated [38]. The latest study investigated cytokines, chemokines, and growth factors in the sera of individuals with dcSSc, and identified a multianalyte signature that could predict the clinical improvement during treatment with imatinib. This indicates the potential for individualized treatment. The authors point out that further research in randomized, placebo-controlled studies is required to validate current results [39]. Ten patients with early dcSSc took part in an open-label, single-group pilot trial which investigated the use of nilotinib TKI. A significant improvement in mRSS was observed. In patients with a decrease in mRSS >20%, expression of the signaling genes of the transforming growth factor beta receptor (TGFBR) and platelet derived growth factor receptor beta (PDGFRB) was significantly higher than in non-improvers. After the treatment, the expression of these genes decreased significantly. Seven out of ten patients were able to tolerate the medication for 12 months. Two patients discontinued the treatment due to mild QTc prolongation. The results of this pilot study require a more conclusive assessment [40]. Nintedanib is a well-known TKI approved for use in treating idiopathic pulmonary fibrosis. Phase 3 of the study on this drug in SSc was completed. The annual decrease in FVC was lower with nintedanib than with placebo. No other clinical benefits were observed. The most frequent adverse effect was diarrhea, which affected up to 75% of patients in the nintedanib group and 31.6% of the patients in the placebo group [41]. Recently, the treatment of SSc-ILD was listed as an indication for nintedanib approved by FDA. An uncontrolled, open-label extension study is underway and will provide long-term data on nintedanib therapy in patients with SSc-ILD. The estimated study completion date is 2022 [42].

### 3.12. IL-6 (Interleukin 6)

Interleukin-6 is involved in the pathogenesis of SSc. Overexpression of IL-6 was observed in the skin and serum of SSc patients, and was correlated with more severe skin involvement and poor long-term survival [43]. Tocilizumab is a monoclonal antibody against the IL-6 receptor. Phase 3 of a clinical trial on the use of tocilizumab in systemic sclerosis has already been completed. No new safety concerns emerged. Safety was coherent with the already known safety profile of tocilizumab. The primary skin fibrosis endpoint was not met. However, FVC results suggest that this treatment might cause stabilization and preservation of lung function as well as maintenance of pulmonary structure in patients with early diffuse SSc-ILD and elevated acute-phase reactants. This treatment could potentially prevent the progression of serious lung complications in the disease [44].

### 3.13. IL-17 (Interleukin 17)

Brodalumab is a biological drug targeting the subunit A of the IL-17 receptor (IL-17RA); thus, it not only inhibits IL-17A but also other members of the IL-17 family. The results of a double-blind, placebo-controlled, phase 3, comparative study of KHK4827 (generic name: brodalumab), with an open-label extension period, in SSc patients who have moderate to severe skin thickening, are promising. In this study, 100 Japanese participants were randomly assigned to one of two groups (the placebo group and the brodalumab group). The change from baseline in mRSS at week 24, which was the primary endpoint, was −16.8 in the brodalumab group (95% CI: −18.7, −14.8) and 4.4 in the placebo group (95% CI: 2.5, 6.4). The difference in change in mRSS between the brodalumab group and the placebo group was −21.2 (95% CI: −23.9, −18.5), indicating a statistically significant decrease in mRSS (*p* < 0.0001). There were no emerging safety issues [45].

### 3.14. Endothelin Receptor

It has been known for many years that systemic sclerosis is characterized by increased levels of plasma endothelin 1 (ET-1); hence, further research on medications affecting endothelin levels is reasonable [46]. Ambrisentan is a selective endothelin A receptor antagonist. It was assessed in two large phase III randomized controlled trials in ARIES-1 and ARIES-2, for the treatment of pulmonary arterial hypertension (PAH). In both studies, ambrisentan demonstrated beneficial effects on PAH symptoms [47]. PAH is a common clinical manifestation in patients with systemic sclerosis; thus, research on the use of this drug in SSc is underway. Another study (phase 2) was performed in Germany which evaluated the effect of ambrisentan on mean pulmonary arterial pressure (mPAP) in patients with SSc and mildly elevated PAH. Progress to SSc-associated pulmonary arterial hypertension was only observed in the placebo group, with none in the ambrisentan group. Ambrisentan was well tolerated. The treatment showed meaningful improvements in the secondary endpoints of cardiac index (CI) and pulmonary vascular resistance (PVR) at rest, but not in the primary endpoint (change in mPAP over 6 months). This indicates the possible benefit of ambrisentan and will help design future trials [48]. Furthermore, initial combination therapy with ambrisentan plus tadalafil was investigated in the AMBITION study, which involved patients with connective tissue disease-associated pulmonary arterial hypertension (CTD-PAH). Reduction in clinical failure events, principally PAH-related hospitalizations, was reported. The combination was well tolerated and considered safe. These data confirm recently revised treatment guidelines, suggesting that initial combined therapy for newly diagnosed patients, even when symptoms are mild, may be of value to clinicians [49].

### 3.15. Autotaxin

Autotaxin is the enzyme responsible for the production of lysophosphatidic acid, which promotes fibrosis in the lungs, kidneys, and liver, and causes the release of pro-inflammatory molecules. GLPG1690 (Ziritaxestat) is a drug that targets and blocks autotaxin. A phase 2a, randomized, double-blind, placebo-controlled, multi-center study was completed, which evaluated the efficacy, safety, and tolerability of orally administered GLPG1690 for 24 weeks in subjects with SSc. GLPG1690 significantly improved mRSS compared to placebo, and was well tolerated when administered along with standard immunosuppressive therapy. This implies a role for autotaxin in the pathogenesis of SSc [50].

## 4. Conclusions

There is an unmet need to discover the processes responsible for the onset of systemic sclerosis. Clarity around this would allow a more effective search for appropriate disease-modifying drugs targeting the molecules strictly involved in the pathogenesis of this disease, e.g., BAFF, JAK, or autotaxin. An additional goal of the research should be to discover how to stop the cascade that leads to molecule production and uncontrolled fibrosis. Nintedanib (TKI) for the treatment of SSc-ILD has, in our opinion, the best chance of quickly entering clinical practice. Imatinib and nilotinib (TKIs), as well as GLPG1690A (autotaxin inhibitor), seem very promising to us, as the improvement in mRSS in their clinical trials was significant. In most clinical trials, the efficacy of treatment was assessed on the basis of changes in the mRSS; however, systemic scleroderma involves different aspects of patients’ everyday life, so new assessment tools could help to adequately assess the therapeutic outcome. A lot of the research on new drugs is promising; however, further investigation remains necessary. Results from large-scale randomized controlled trials will provide the most reliable data.

## Figures and Tables

**Table 1:** Characteristics of mentioned clinical trials (drug, target, phase, type of study, dates of last update, and study completion).

Drug Name	Target	NCT Number	Phase	Type of Study		Last Update Posted	Study Completion Date	Actual/Estimated
Abatacept	CD80/CD86	NCT02161406	II	Randomized	double-blind, placebo-controlled	February, 2020	October, 2018	Actual
MEDI-551	CD19	NCT00946699	I	Randomized	Double-blind placebo-controlled	November, 2014	March, 2014	Actual
Rituximab	CD20	NCT04274257	II	Randomized	Double-blind placebo-controlled	February, 2020	November, 2019	Actual
Rituximab/Belimumab	CD20/BAFF	NCT03844061	II	Randomized	double-blind, placebo-controlled	September, 2020	February, 2022	Estimated
Brentuximab Vedotin	CD30	NCT03222492	I/II	Randomized	double-blind, placebo-controlled	November, 2020	January, 2023	Estimated
Brentuximab Vedotin	CD30	NCT03198689	II	Randomized	unblinded, uncontrolled	May, 2019	July, 2021	Estimated
Iguratimod	TNF	NCT04515706	-	Randomized	high level double-blind	August, 2020	January, 2024	Estimated
Fresolimumab	TGF-β	NCT01284322	I	Not Applicable	open label	July, 2014	March, 2014	Actual
Belimumab	BAFF	NCT01670565	II	Randomized	double-blind, placebo-controlled	March, 2017	February, 2016	Actual
Sar100842	LPAR1	NCT01651143	II	Randomized	double-blind, placebo-controlled	March, 2016	April, 2016	Actual
Riociguat	sGC	NCT02915835	II	Randomized	double-blind, placebo-controlled	September, 2019	July, 2018	Actual
Riociguat	sGC	NCT02283762	II	Randomized	double-blind, placebo-controlled	February, 2020	March, 2019	Actual
Tofacitinib	JAK	NCT03274076	I/II	Randomized	double-blind, placebo-controlled	May, 2020	November,2019	Actual
Nilotinib	JAK	NCT01166139	II	Open-label	uncontrolled	October, 2017	January, 2015	Actual
Imatinib	JAK	NCT00555581	II	Open-label	uncontrolled	February, 2018	December, 2011	Actual
Nintedanib	JAK	NCT03313180	III	Open-label	uncontrolled	December, 2020	December, 2022	Estimated
Nintedanib	JAK	NCT02999178	III	Randomized	double-blind, placebo-controlled	May, 2020	August, 2019	Actual
Peficitinib	JAK	NCT05177471	-	-	observational Study	January, 2022	April, 2022	Estimated
Tocilizumab	IL-6	NCT02453256	III	Randomized	double-blind placebo-controlled	March, 2020	February, 2019	Actual
Brodalumab	IL-17	NCT04368403	I	Open-label	uncontrolled	June, 2021	December, 2022	Estimated
Ambrisentan	Endothelin receptor	NCT02290613	II	Randomized	double-blind placebo-controlled	April, 2020	December, 2017	Actual
GLPG1690	autotaxin	NCT03798366	II	Randomized	double-blind, placebo-controlled	May, 2021	June, 2020	Actual

CD28-CD80/86; CD19 (cluster of differentiation 19); CCL24 (chemokine c-c motif ligand 24, eotaxin 2); CD20 (cluster of differentiate 20); CD30 (TNFRSF8—tumor necrosis factor receptor superfamily member 8); TNF (tumor necrosis factor); TGF-β (transforming growth factor β); BAFF—B-cell activating factor; LPA1 receptor—lysophosphatidic acid receptor 1; sGC—soluble guanylate cyclase; JAK—Janus kinases; IL-6 (interleukin 6); IL-17 (interleukin 17).

## Data Availability

M.M., A.B., K.K.P. was responsible for the ideation; A.B. and K.K.P. performed the literature search and data analysis; M.S., R.A.M., J.C., L.R. drafted and revised the work.
